# Cryptic *Plasmodium* chronic infections: was Maurizio Ascoli right?

**DOI:** 10.1186/s12936-020-03516-x

**Published:** 2020-11-30

**Authors:** Wuelton Monteiro, José Diego Brito-Sousa, Aleix Elizalde-Torrent, Camila Bôtto-Menezes, Gisely Cardoso Melo, Carmen Fernandez-Becerra, Marcus Lacerda, Hernando A. del Portillo

**Affiliations:** 1 Fundação de Medicina Tropical Dr Heitor Vieira Dourado (FMT-HVD), Manaus, Amazonas Brazil; 2 Universidade Do Estado Do Amazonas (UEA), Manaus, Amazonas Brazil; 3 ISGlobal, Hospital Clínic—Universitat de Barcelona, Barcelona, Spain; 4 Institut D’Investigació Germans Trias I Pujol (IGTP), Badalona, Barcelona Spain; 5 IrsiCaixa AIDS Research Institute, Badalona, Catalonia Spain; 6 Instituto Leônidas & Maria Deane (ILMD), Fiocruz, Manaus, Amazonas Brazil; 7 Institució Catalana de Recerca I Estudis Avançats (ICREA), Barcelona, Spain

**Keywords:** Ascoli‘s method, Cryptic infection, *Plasmodium*, Parasite recurrence, Spleen

## Abstract

Cryptic *Plasmodium* niches outside the liver possibly represent a major source of hypnozoite-unrelated recrudescences in malaria. Maurizio Ascoli, an Italian physician and scientist, suggested that infection was maintained as a result of the persistence of endoerythrocytic parasites in the circulatory bed of some internal organs, mainly the spleen. This would explain a proportion of the recurrences in patients, regardless of the *Plasmodium* species. Ascoli proposed a method that included the co-administration of adrenaline, in order to induce splenic contraction, and quinine to clear expelled forms in major vessels. Driven by controversy regarding safety and effectiveness, along with the introduction of new drugs, the Ascoli method was abandoned and mostly forgotten by the malaria research community. To date, however, the existence of cryptic parasites outside the liver is gaining supportive data. This work is a historical retrospective of cryptic malaria infections and the Ascoli method, highlighting key knowledge gaps regarding these possible parasite reservoirs.

## Hypnozoites, the prototype of cryptic chronic infections

The possible existence of malaria parasites outside red blood cells (RBCs) has been considered since the very beginning of malariology [1–3]. It is related to the approximate ten-day period in which parasites disappear from blood after infections. The discovery of the exoerythrocytic forms of the malaria parasites in the liver of experimentally infected nonhuman primates and later in human livers established that malaria parasites could invade cells other than RBCs. Yet, it took years to unveil the mystery of the long-term prepatent periods observed in temperate strains of *Plasmodium vivax*. In fact, the discovery of a dormant exoerythrocytic stage, the hypnozoite (from the Greek words *hypnos*—sleep—and *zoon*—animal), a term coined in relation to studies on other non-plasmodial apicomplexan parasites [4], was made only in 1982. This happened as a result of a collaboration between Krotoski in the USA and Garnham in the UK. They attributed the phenomenon of extended prepatent periods and periodic reappearance of microscopically detectable *P. vivax* parasites in the bloodstream to an activation of quiescent hepatic hypnozoites [5].

Malaria relapses occur at variable times after infection and represent a major hurdle in malaria epidemiology [6]. A relapse is thought to exclusively originate from quiescent intracellular hypnozoite liver stages and in humans it is caused by *P. vivax* and *Plasmodium ovale*. This biological characteristic makes *P. vivax* a major cause of ongoing febrile illness in endemic areas of Asia, Central and South America, and the horn of Africa [7]. Strains of *P. vivax* from different geographical areas are thought to exhibit varied relapse timing. In tropical regions, strains relapse quickly (3–6 weeks), whereas those in temperate regions do so more slowly (6–12 months) [8, 9]. Activation of heterologous hypnozoites is the most common cause of the first relapse episodes in patients with vivax malaria [10]. However, the epidemiology of the different relapse phenotypes, including factors associated with the triggering of relapses, is not adequately known despite the potential for sustained transmission in such a diverse range of environmental niches; thus representing an important knowledge gap for malaria control and elimination.

The achievement of malaria eradication is thought to be unlikely without the development of effective hypnozoiticides. Primaquine is routinely used to prevent relapses of *P. vivax* and *P. ovale* malaria and has played a prominent role in several successful elimination campaigns. The long-acting tafenoquine is a recently FDA-approved 8-aminoquinoline, which opens venues to an easier radical cure regimen with a single dose [11]. The availability of in vitro* Plasmodium* liver-stage models and the possibility of using humanized mouse models engrafted with functional human cells and tissues could facilitate understanding of *Plasmodium* liver-stage biology, further enabling the search for compounds that kill hypnozoites or disrupt their quiescence [12–14].

## Non-hypnozoite persistence of *Plasmodium*

The existence of non-hypnozoite stages lasting longer than the acute malarial disease has been suggested in several studies. In fact, in endemic areas it is not unusual to find *Plasmodium*, including non-relapsing species, in the peripheral blood of persons who remain asymptomatic for long periods of time. For instance, although *Plasmodium malariae* is thought to lack the ability to relapse from dormant liver stages, it is well known for possessing a capacity for “dormancy”, lasting for decades in a well-documented clinical case [15]. Moreover, parasites somewhere in the body can give rise to clinical *Plasmodium falciparum* malaria up to at least a decade after people have left endemic regions [16–19]. Cryptic *P. falciparum* infection is also related to transfusion-transmitted malaria in non-endemic areas, transferred from asymptomatic individuals years after they were in a malarious area [19–21]. Shanks [22] suggests that *P. falciparum* maintains itself over extended periods of time within human blood and bone marrow in order to bridge periods between transmission times. Moreover, a hypothesis compatible with the continuity concept of biological evolution has been proposed, in which relapse may potentially be caused by all *Plasmodium* spp. [23]. According to this theory, differences between the various *Plasmodium* spp. are quantitative rather than qualitative. *Plasmodium vivax* causes relapses most frequently, whereas other species, such as *P. ovale* and sometimes *P. malariae* do so less often, and *P. falciparum* rarely causes relapses [23].

Some evidence of merozoite sequestration and persistence as cryptic infections in extrahepatic and non-bloodstream sites can also be found in the literature. Although prompt treatment of women with peripheral parasitaemia during pregnancy limits placental pathology [24], a study revealed a nidus of malaria-infected erythrocytes that was identified in a single intervillous space in placental tissue from a woman who had no other evidence of peripheral or placental blood parasitaemia, suggesting that *P. falciparum* can persist as a dormant blood-stage form [25]. In addition, dormant blood ring stages have been reported for artesunate-treated parasites in vitro [26, 27]. Furthermore, high prevalence and abundance of gametocytes of *P. falciparum* occur in the bone marrow [28]. Interestingly, the majority of immature stage II to IV gametocytes were observed in extravascular spaces [29]. Histological studies revealed a niche in the extravascular space of the human bone marrow where gametocytes formed in erythroid precursor cells and underwent development before re-entering the circulation [30].

In the case of *P. vivax*, even though the presence of parasites in the bone marrow was first acknowledged in 1894 [31], studies of parasites in this tissue were scarce and somewhat contradictory. Thus, external punctual evaluation and nuclear abnormalities in erythroblasts were reported in vivax infections [32, 33]. Yet, an in-depth ultrastructure analysis of bone marrow aspirates from children with severe anaemia failed to detect parasites in this tissue [34]. More recently, unequivocal evidence of the presence of *P. vivax* in the bone marrow was reported in a clinical case with both gametocytes and schizonts readily detected [35]. In addition, experimental *P. vivax* infections in splenectomized monkeys also revealed that the bone marrow is a niche for parasites during active infections [36]. In splenectomized patients, parasitaemias also tend to be higher and patients suffer from more severe disease [37].

Non-bloodstream sites of parasite occurrence have also been described in *Plasmodium berghei* and *Plasmodium yoelii* infections in mice, where a small fraction of the sporozoite inoculum remains in the skin and begins to develop into exoerythrocytic forms not eliminated by primaquine and which can persist for days [38]. Therefore, an analysis of malaria in rodents shows us that the skin is not just the route to the liver, but it is also the final destination for many inoculated parasites, where they can differentiate into merozoites and possibly persist [39, 40]. Moreover, in *Plasmodium chabaudi* experimental infections, dendritic cells were shown to hold infective parasites capable of inducing new infections upon injection into naïve mice [41]. Adherence of *P. berghei* within the spleen has also been demonstrated [42, 43]. Altogether, these studies suggest that malaria parasites could remain hidden in cryptic niches during chronic infections. Whether such hidden parasites are metabolically active or not is a major concern, given that more active parasites would be a better target for radical treatment.

## Cryptic *Plasmodium* infection in the spleen

The spleen is a complex organ that is perfectly adapted to selectively filtering and destroying senescent RBCs, infectious microorganisms and *Plasmodium*-parasitized RBCs during malaria attacks [44, 45]. In fact, infection by *Plasmodium* is the most common cause of splenomegaly, albeit variably. Splenic enlargement is a common clinical characteristic of malaria, estimated to occur in 70–80% of acute cases with the size of the spleen generally normalizing after anti-malarial treatment [46]. In areas of stable transmission spleen enlargement is noticeable in 50–80% of individuals, being directly associated with acquisition of immunity, as shown by its higher prevalence in children and correlation with the host immune response [47].

Evidence of splenic exoerythrocytic reproduction of *Plasmodium* was first seen in avian malaria. MacCallum, in 1898, observed developmental stages of *Plasmodium relictum* in the spleen of infected birds [48]. By the late 1930s all studied avian malaria parasites had been reported to have a phase of multiplication in various nucleated cells before (and after) parasites appeared in the blood [49].

In the early twentieth century, experiments suggested a role for the human spleen as a reservoir of RBCs (and other ‘figured elements’ of the blood), establishing the known theory of erythrocyte reservoirs in the spleen [50, 51]. According to this theory, RBCs are stored in the spleen and released as a result of splenocontraction stimulated by the nervous system, forcing this blood into the general circulation. This fact, coupled with results on the dissimilar histotropic behaviour of *Plasmodium* strains [52, 53], the spleen being a target organ of these parasites, led to the reasoning that malaria could only have its radical cure achieved after the expulsion of all parasites possibly harboured in this organ, that is, solving splenomegaly completely.

**Fig. 1:**
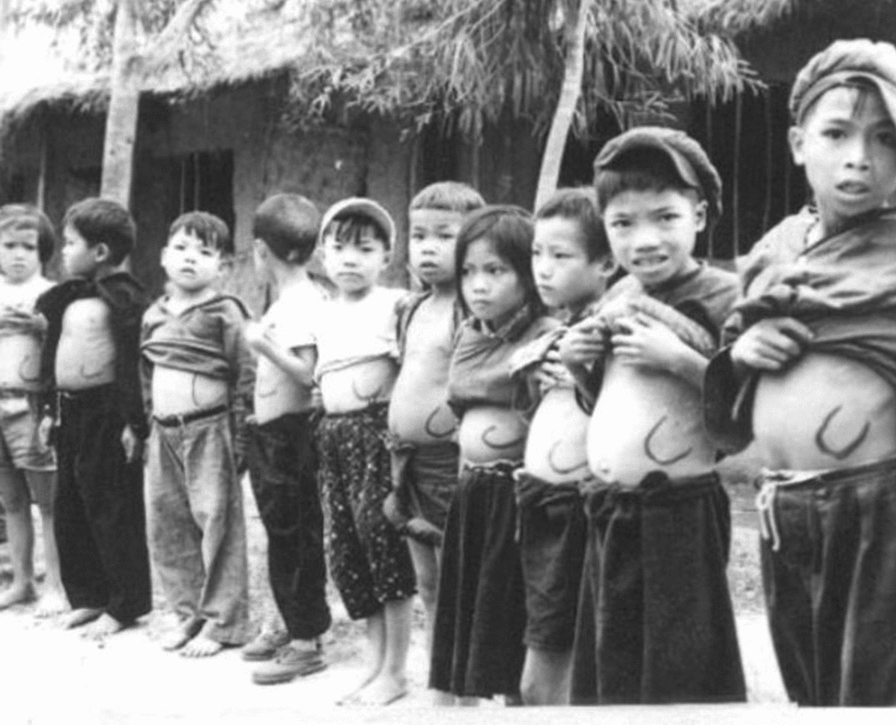
The Hackett’s index. Visual example of the use of the splenic rate as a follow-up measure for patients with hyper-reactive malarial splenomegaly syndrome

Indirect evidence of human malaria infections in the spleen has been suggested by clinical observations of the Hackett’s index, the hyper-reactive malarial splenomegaly syndrome (HMS) and clinical cases of untreated spontaneous spleen ruptures. Firstly, for epidemiological studies, the average size of enlarged spleens was a very helpful malariometric index (Hackett’s index), which was calculated from the frequency distribution of five classes of enlarged spleen and ranged from non-palpable on deep inspiration to lowest point palpable in the right iliac fossa [54]. Since spleen size showed a positive linear relationship with parasite infection rates, this classification was used to decide upon the deployment of control efforts (Fig. 1) [55]. Secondly, HMS, a life-threatening condition caused by chronic antigenic stimulation, was attributed to malaria parasites [56]. In fact, it was clinically useful to monitor spleen size during the course of malaria infection, because if the spleen failed to regress in size as expected after treatment, there was a greater likelihood of recurrences [57]. Establishing the relationship may appear straightforward, but this could also be confounded by intensity of transmission and, therefore, higher risk of new vivax episodes and/or recurrences. Thirdly, the presence of malaria parasites in the spleen was reported in a clinical case of spleen rupture in a non-treated *P. vivax* patient [58]. More recently, parasite DNA in spleen sections from another splenic rupture case due to *P. vivax* infection was reported. Histological analyses revealed the presence of large numbers of intact *P. vivax*–infected RBCs in the splenic cords [59].

## The Ascoli method

**Fig. 2:**
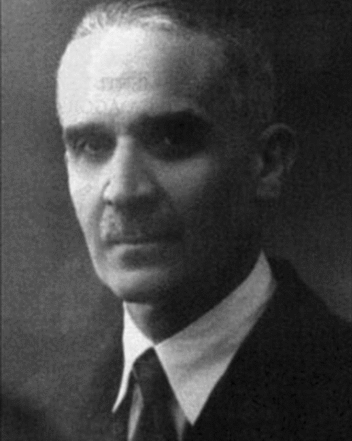
Maurizio Ascoli (Trieste, July 14, 1876 to Palermo, August 4, 1958). Source: https://it.wikipedia.org/wiki/Maurizio_Ascoli

What happens in spleens of humans seemed clarified by the clinical observations of the Italian physician Maurizio Ascoli (Trieste, July 14, 1876 to Palermo, August 4, 1958) (Fig. 2 and Box 1). From a historical perspective, in the 1920s a wide variety of agents were tried in order to mobilize latent parasites thought to be responsible for malarial recurrences in humans. These included small doses of quinine, strychnine, adrenaline, exposure to higher altitude and two doses of typhoid-paratyphoid vaccine, rationalizing that using strychnine or adrenalin has similar effects [60, 61].

**Box 1:** Brief biography of Maurizio Ascoli

Born July 14, 1876, Triest
In 1899, Ascoli graduated from Medical School in Turin, where he was a student of Bizzozzero. To complete his scientific training, he went to Germany, where he knew his friend Paul Ehrlich. Back in Italy, he continued his studies under the guidance of Luigi Devoto
In 1904, Ascoli obtained a teaching qualification in special medical pathology and from 1907 to 1910 he had the task of teaching this discipline at the University of Pavia
During the years of the First World War, Ascoli served in the operations area and reached the rank of Lieutenant Colonel
From 1920 to 1921, Ascoli taught medical pathology at the University of Palermo
In 1927, Ascoli became the owner of a medical clinic in Catania
In collaboration with Vincenzo Mutolo, he devised the simultaneous bilateral pneumothorax method for the treatment of tuberculosis
In 1929, Ascoli was called to the chair of Medical Clinics at Palermo
During the 1930s, Ascoli described his ‘*humoral*’ treatment of chronic malaria associated with recurrences, splenomegaly, anemia, and cachexia by intravenous administration of adrenaline
At the beginning of 1939, after the promulgation of the Racial Laws in Italy, he was evicted from the Medical Clinics at Palermo, due to his Jewish origins
In 1943, he was reinstated in teaching
After leaving teaching in 1951 when he exceeded the employment age limit, Ascoli devoted himself for a time to the management of Palermo’s Cancer Hospital

Ascoli method was based on a malaria pathophysiology mechanism, reproducing the natural course of the disease in a fast-paced and abbreviated form with the same defenses which the body uses [62]. It was not unusual for some individuals with afebrile chronic malaria to experience a typical bout of fever after a violent emotion, trauma, haemorrhage or a cold bath. A review of the literature to discover the factual basis of such varied reports, led to the conclusion that it seemed likely that human stress sometimes induced recrudescence of an otherwise asymptomatic infection [21]. These episodes showed peculiar characteristics that can be summarized as follows: (1) they manifested as an isolated and ephemeral attack; (2) they relied on an occasional cause; (3) they appeared a few hours after the abrupt cause; (4) they generally disappeared without medical treatment [61, 62]. Based on these observations, it would seem that mature parasite sequestration occurred somewhere in the human host, being a source of febrile paroxysms after parasites enter circulation. The maturation and release of parasites occurred in the same way, although with less dramatic symptoms, but with the same adverse effects for the patients [62]. Where do these parasite deposits occur that can suddenly enter into the bloodstream? Ascoli thought that they were present as a splenic reservoir, far away from the bloodstream and in which the parasites, sheltered from the harmful effects of drugs and antibodies, multiplied comfortably building true cryptic foci [62]. This exoerythrocytic cycle, according to Ascoli, was the *conditio sine qua non* for explaining the maintenance of the infection [61].

On the basis of Ascoli’s work, evolution of malarial infection may be distinguished by two phases. The first is a septicemic phase responsible for the typical clinical manifestations in which the parasites mature in circulating RBCs, whereas the second is a focal phase with parasites held in splenic foci, the patient is asymptomatic and infection becomes chronic. Against the septicemic phase, the host puts into action a specific immune response elicited by the plasmodial infection. However, the schizonticidal effect of this immune response would be negligible in the splenic foci because of parasite exclusion from the blood circulation. For the same reason, anti-malarial drugs administered to the patient seemed not to reach these splenic parasites. Local defense was instead restricted to the adrenal hormone that increased the tone of the spleen, leading to its contraction, which reduced the extent of the morbid foci by evicting parasites into the bloodstream. A malaria attack during the latency period may also be provoked by the intravenous injection of adrenaline resulting in splenic contractions [61]. In the absence of activating factors, parasites would have continued living and multiplying, hidden in splenic foci in clinical silence, well balanced with the host defenses.

In their publications, Ascoli and colleagues presented results for a series of patients with chronic malarial splenomegaly, in whom the splenomegaly had resisted the usual quinine-arsenical treatments. The patients received further intravenous injections of minimal doses of adrenaline during the afebrile stage. Of particular importance was the fact that the doses of adrenaline used were considered small, beginning with 0.01 mg and finally reaching a total of 2, 3, and, rarely, 4 mg [64, 66]. By the subcutaneous method, contraindicated according to Ascoli, the equivalent dose of the drug (in the proportion of 1:100) would amount to the large quantity of up to 400 mg [63]. The injections were given daily or on alternative days if the patient’s general reaction was intense, the dosage increased by 0.01 mg each administration until the total quantity was reached. The final (maximum) dose of 0.1 mg was repeated about twenty or thirty times until the swelling of the spleen disappeared, usually within two months [63, 64].

During the course of the treatment, renewal of the fever was often observed, ranging from a slight increase in temperature to severe febrile episodes. Reactivation, usually during the second week of treatment, could be early or late, occurring at the beginning, the end, or even a few days after the cessation of treatment. In these cases, or when treatment began during the febrile period, clinicians added the administration of low doses of quinine for up to two days after reaching apyrexia. By subjecting to this treatment a cases series of 10 recurrent and chronic malaria patients and by administering to them no more than 2–4 mg of adrenaline, Ascoli reported: 1. cessation of transient febrile events; 2. total or partial reduction of splenic hypertrophy with cessation of splenic pain; 3. significant improvement in regards to anaemia and leucopenia; 4. weight gain; 5. well-being rapidly restored, improvement in the general status, disappearance of the ‘malarial facies’; and 6. negative Henry’s melanoflocculation reaction [63]. Although these results were not uniform, those authors deduced the utility of treatment with adrenaline associated with, not in substitution of, quinine during the febrile periods of the malarial attack. It was thought that adrenaline enhances the effect of quinine and makes the anti-malarial treatment at sub-therapeutic doses effective. This phenomenon was explained by the assumption that, thanks to the splenocontraction caused by adrenaline, quinine can act on almost all the parasitized RBCs, whereas otherwise the quantities of blood stored in spleen reservoirs would escape its influence [65]. It was also observed that adrenaline triumphed over the relative quinine-resistance that was so frequently encountered in the tropics in individuals taking malarial chemoprophylaxis [64].

**Fig. 3:**
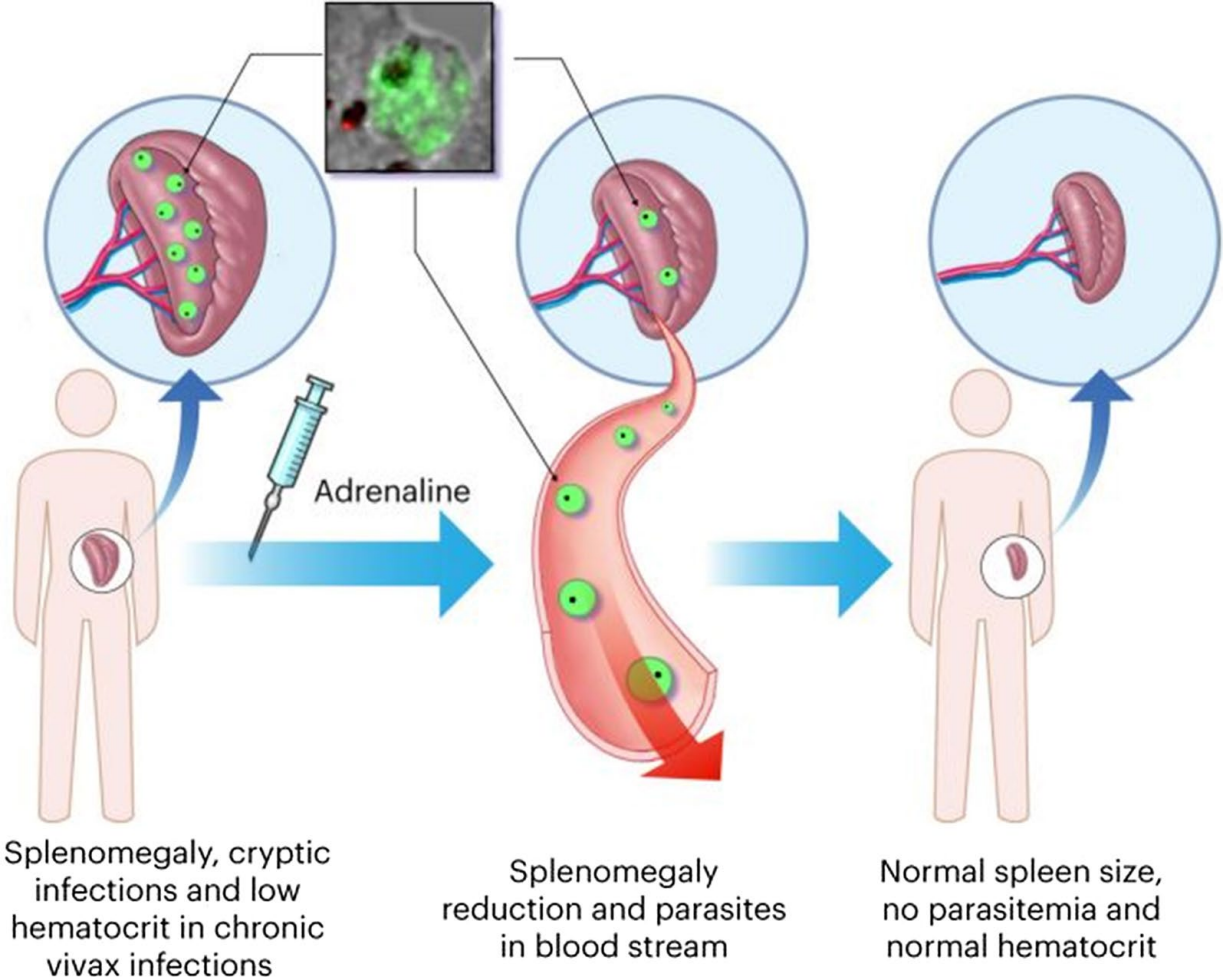
Cryptic *Plasmodium* infections. The scheme represents potential dormant *Plasmodium* niches in the liver (*P. vivax* hypnozoites), as well as in the bone marrow and the spleen (*Plasmodium*-infected red blood cells). In the upper left square, anti-vir immuno-histochemistry of a spleen section with stained *P. vivax* parasites is shown. According to Ascoli’s theory, after the injection of adrenaline parasites were released in the peripheral circulation. After treatment, the spleen returns to its normal size, parasitaemia is no longer seen and the haematocrit increases back to normal levels

The time interval that elapsed between the end of treatment and recurrence varied notably. Some data point to an inverse correlation between time interval and the duration of infection [66]. Pietro Riolo reported the results of treatment of 18 cases of chronic malaria with splenomegaly by intravenous injections of adrenalin, stating that 15 of the 18 patients were followed up [67]. Nearly two years after the termination of treatment no relapses had been observed and spleen size remained within the limits noted at the end of treatment. According to the same author, the treatment with adrenaline plus quinine of 20 chronic malaria cases presenting with frequent relapses secured complete amelioration during such a febrile recurrence. The patients were kept under observation for a year, at the end of which only one patient had a spleen that was palpable. Regarding malarial anaemia, a study of six malaria patients suggested a maximum benefit of Ascoli method when the spleen size reduction was complete and no or little effect on haemoglobin levels when there was no reduction [63], indicating that the mode of action may be the suppression of factors inducing anaemia. Indeed, the first functional explorations of the spleen in malaria patients showed splenic contraction occurring after adrenaline administration, with coincident erythrocytosis and haematocrit improvement [68]. Ascoli alleged that severe malaria is a consequence of retention of parasitized RBCs in the capillaries of the internal organs, partly caused by vascular hypotonia due to adrenal insufficiency, and which are real extra-splenic malarial nests excluded from the circulation, barely accessible to anti-malarials. If adrenaline is administered in such cases, subsequent vasoconstriction results in the reversal of capillary blockage, restoring the microcirculation in respect of the small vessels of the internal organs, and leading to recovery from the severe consequences of vascular obstruction (Fig. 3).

## Practical aspects of the therapy of chronic malarial splenomegaly and Ascoli’s clinical experiments

Outside Italy, the efficacy of Ascoli’s method became known worldwide, culminating in the inclusion of the treatment in the 1970s in important textbooks on tropical diseases, especially for cases of malarial splenomegaly and cerebral malaria [69–71]. According to these guidelines, as regards to cerebral malaria, adrenaline would cause vasoconstriction of the peripheral blood vessels, particularly those in the brain, and contraction of the spleen. The parasites driven out from the peripheral vessels would then be more safely destroyed by quinine in the larger vessels, where their destruction would not block the vital organs. Adrenaline also reverses the state of shock, which is a feature of this condition, by introducing exogenous adrenaline and not taxing the patient's adrenals. Positive field experiences were reported from Spain [72], Albania [73], Algeria [74], Brazil [75], Bangladesh [76, 77], India [78, 79], Sudan [80] and by the US army [81, 82] in managing cerebral malaria, algid malaria and malarial splenomegaly. In Goa, India, Froilano de Mello pointed out in 1938 the number of injections required and the unpleasant, sometimes serious, symptoms observed, not recommending the method as advisable for mass treatment, but encouraging it in hospital practice, provided the unpleasant reactions are avoided [78].

Unfortunately, the effectiveness of the method in patients infected with different *Plasmodium* species is uncertain. In some cases of both chronic vivax and falciparum malaria presenting with frequent recurrences, the febrile episodes were completely resolved [67]. In the Brazilian Amazon, Djalma Batista, a prestigious physician in Manaus, treated 34 individuals with a previous history of malaria by Ascoli method and reported in 1946 that 16 cases became microscopically positive during the follow-up (14 *P. vivax*, 1 *P. falciparum* and 1 mixed infection) [75]. It is one of the few reports in which the unsuccessful cases are also reported, which suggests that some publication bias might have occurred in other reports. In Italian East Africa, where *P. vivax* prevails amongst Duffy-positive population, an impressive reduction in mortality was attributed to quinine-adrenaline treatment, with a further decrease in the incidence in malaria severity [65]. In Vietnam, the adrenaline provocative test was of no diagnostic value in 18 recrudescent cases of falciparum malaria [83], an experience further confirmed by others [84, 85]. Although common in both *P. falciparum* and *P. vivax* endemic areas, spleen enlargement seems to be more pronounced in the latter infection [86], thus suggesting greater usefulness of the method for vivax malaria.

Safety concerns led to strong debates among clinicians early in the adoption of the treatment worldwide [87]. The action of the drug may be said to be immediate, and the side effects include pallor, headache, tachycardia or arrhythmia, tremors, sometimes psychic and motor excitation, and palpitations. Hypotension, collapse, and even sudden death were well-known sequelae when intravenous quinine injections were given to seriously ill patients [88]. From the 1970s, the rejection of Ascoli method became very intense because of safety and efficacy considerations, particularly in hyper-endemic falciparum areas in Africa [83, 84, 88]. For critics, adrenaline-induced arteriolar constriction resulting in driving parasites out from the peripheral vessels, and splenic contraction altering the distribution of parasitized cells within the vascular system are biologically plausible, but their clinical demonstration was never actually achieved [89]. With changes in the first-choice treatment for malaria, there was no justification for the use of adrenaline as an agent for the prevention and treatment of the hypotension caused by the intravenous injection of quinine. Finally, following acceptance of the hypnozoite explanation for relapsing malaria [5], malaria researchers lost interest in investigating other tissular exoerythrocytic forms, mostly but not entirely [90] considered to be of secondary importance in the life cycle of *Plasmodium*.

## Concluding remarks

Asymptomatic carriers of malaria parasites are a major challenge for malaria elimination. The old and new evidence reviewed here indicates that cryptic niches outside the liver, in particular in the spleen, can represent a major source of hypnozoite-unrelated recrudescences. It is noteworthy, however, that there is no direct evidence that adrenaline disrupts the spleen in a manner that redistributes the anatomic locations of any parasites residing in them. Furthermore, no information on the impossibility of quinine to reach spleen niches as much as the peripheral blood is available. Thus, the results of the Ascoli method should be interpreted with caution. Nevertheless, they indicated the spleen as a major parasite reservoir and new evidence is indicating that parasites are able to create cryptic infections in this organ as well as in the bone marrow. Moreover, in cases of non-relapsing malaria, evidence from human infections and animal models indicates that these parasites have also evolved cryptic niches that are associated with chronic infections. The source of such recrudescent parasites is controversial and many key knowledge gaps concerning cryptic niches remain to be addressed (Box 2). In addition, parasites in such niches seem sheltered from the actions of drugs and antibodies. Thus, a renewed interest in re-investigating cryptic non-hypnozoite *Plamodium* niches during asymptomatic chronic infection is warranted. Whether Ascoli’s theory was right is difficult to determine because it was never tested in experimental models, and clinical trials nowadays would be considered unethical due to the severe side effects of adrenaline. Furthermore, there might have been a bias toward publication of only positive experiences. However, all data generated by this worldwide used technique could provide useful insight into cryptic *Plasmodium* stages, particularly *P. vivax*, which might have therapeutical implications when pursuing elimination in the field. As stated by Aluízio Falcão Filho, the Brazilian writer, in his novel ‘The journalist, the writer and the aviator’: ‘*The past is unpredictable’*.

**Box 2:** Outstanding research questions of cryptic *Plasmodium* chronic infections

Are cryptic asexual blood stages during chronic infections hidden at plain sight?
Do they represent the main source of parasites during asymptomatic infections?
Are the bone marrow and spleen the main places for cryptic chronic infections?
Is there host/parasite intercellular communication facilitating establishment of cryptic niches?
What selective advantages are behind the evolution of cryptic hypnozoite dormant stages *vs* cryptic active stages in *P. vivax*?
What is the factual degree of sheltering of these cryptic niches, both in terms of evasion of immune responses and anti-malarial drugs?
What are the implications of these sheltered niches for malaria elimination?

## Data Availability

Not applicable.

## Abbreviations

FDA: Food and Drug Administration
HMS: Hyper-Reactive Malarial Splenomegaly Syndrome
RBC: Red Blood Cell
